# Spatial Regulation of Root Growth: Placing the Plant TOR Pathway in a Developmental Perspective

**DOI:** 10.3390/ijms160819671

**Published:** 2015-08-19

**Authors:** Adam Barrada, Marie-Hélène Montané, Christophe Robaglia, Benoît Menand

**Affiliations:** 1 Laboratoire de Génétique et Biophysique des Plantes, Aix-Marseille Université, Marseille F-13009, France; E-Mails: adam.barrada@etu.univ-amu.fr (A.B.); marie-helene.montane@univ-amu.fr (M.-H.M.); christophe.robaglia@univ-amu.fr (C.R.); 2 Centre National de la Recherche Scientifique (CNRS), Unité Mixte de Recherche Biologie Végétale & Microbiologie Environnementales, Marseille F-13009, France; 3 Commissariat à l’énergie Atomique et aux Energies Alternatives (CEA), Institut de Biologie Environnementale et Biotechnologie (IBEB), Marseille, F-13009, France

**Keywords:** cell growth, proliferation, elongation, meristem, root, plant, auxin, cytokinin, environment, TOR (target of rapamycin)

## Abstract

Plant cells contain specialized structures, such as a cell wall and a large vacuole, which play a major role in cell growth. Roots follow an organized pattern of development, making them the organs of choice for studying the spatio-temporal regulation of cell proliferation and growth in plants. During root growth, cells originate from the initials surrounding the quiescent center, proliferate in the division zone of the meristem, and then increase in length in the elongation zone, reaching their final size and differentiation stage in the mature zone. Phytohormones, especially auxins and cytokinins, control the dynamic balance between cell division and differentiation and therefore organ size. Plant growth is also regulated by metabolites and nutrients, such as the sugars produced by photosynthesis or nitrate assimilated from the soil. Recent literature has shown that the conserved eukaryotic TOR (target of rapamycin) kinase pathway plays an important role in orchestrating plant growth. We will summarize how the regulation of cell proliferation and cell expansion by phytohormones are at the heart of root growth and then discuss recent data indicating that the TOR pathway integrates hormonal and nutritive signals to orchestrate root growth.

## 1. Introduction

Many studies on a molecular and a cellular scale have allowed a better understanding of the mechanisms involved in the establishment of the different tissues during plant development. Tissues grow in a well-organized fashion which shapes the plant’s final structure. Plant growth is the result of two main cellular mechanisms, proliferation and elongation, which are coordinated by phyto-hormones and modulated by environmental factors like nutrients or light. How these hormonal and environmental signals are integrated into plant developmental processes is not yet fully understood. The primary root with its three distinct zones (meristematic zone, elongation zone and maturation zone), each representing a different cell growth stage, follows a well-defined developmental pattern offering a good model for the study of cell growth regulation. Recent studies have shown that the conserved TOR (target of rapamycin) signaling pathway controls root growth [1,2,3]. The TOR kinase is a key regulator of cell growth and proliferation in animals and yeast and is emerging as a central regulator of environmental and hormonal responses in plants.

This review will focus on primary root growth. The mechanisms that establish root patterning during embryo and post-embryonic development [4,5], as well as initiation of lateral roots [6], will not be considered in this review. Rather the main post-embryonic steps and effectors of root growth will be taken as simplified platform to discuss the connection of the TOR signaling pathway with hormonal and nutritional status, cell cycling state and post-mitotic cell expansion. We will summarize how a complex regulation of cell proliferation and expansion is involved in the establishment and maintenance of the longitudinal root growth and how these processes are coordinated by two essential hormones, auxin and cytokinin. We will then discuss how the evolutionary conserved TOR pathway could integrate hormonal and nutritive factors to regulate root growth.

## 2. Primary Root Morphology and Zonation Is the Result of Controlled Cell Proliferation, Differentiation and Expansion

### 2.1. Root Patterning and Zonation

#### 2.1.1. Radial Patterning

Most vascular plants grow in a bidirectional and polarized manner as their cells increase in number in the shoot and root meristems and in size in the elongation zones. Two main cellular mechanisms can then be distinguished: proliferation, which consists of an increase in cell number inside an organ through doubling the cellular content coupled with mitosis leading to daughter cells; and post-mitotic expansion (also called enlargement or elongation), which consists of a considerable increase in size through vacuolization and cell wall expansion of non proliferating cells.

**Figure 1:**
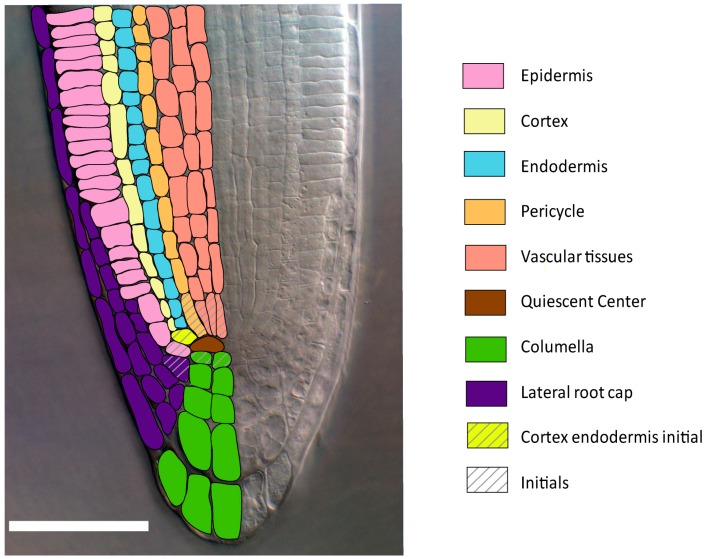
Root radial patterning of the model flowering plant *Arabidopsis thaliana.* Differential interference contrast microscopy picture of the *A. thaliana* primary root tip. Bar 50 µm. Each color corresponds to a different cell layer.

In the *Arabidopsis thaliana* primary root, cells originate from stem cells called initials that are organized around the quiescent center (QC) that is made of undifferentiated cells that divide very rarely. These initials form a slowly dividing stem cell reservoir of cells that have already acquired an identity during embryogenesis [7,8]. Division of initials results in the formation of distinct cell layers (or tissues) concentrically arranged around the longitudinal axis (Figure 1). These include the stele, which contains the vascular tissues, the pericycle, the endodermis, the cortex, and the epidermis. The columella and the lateral root cap provide additional layers below the quiescent center. Each of these cell types expresses specific cell identity regulators [4].

#### 2.1.2. Longitudinal Root Zonation

**Figure 2:**
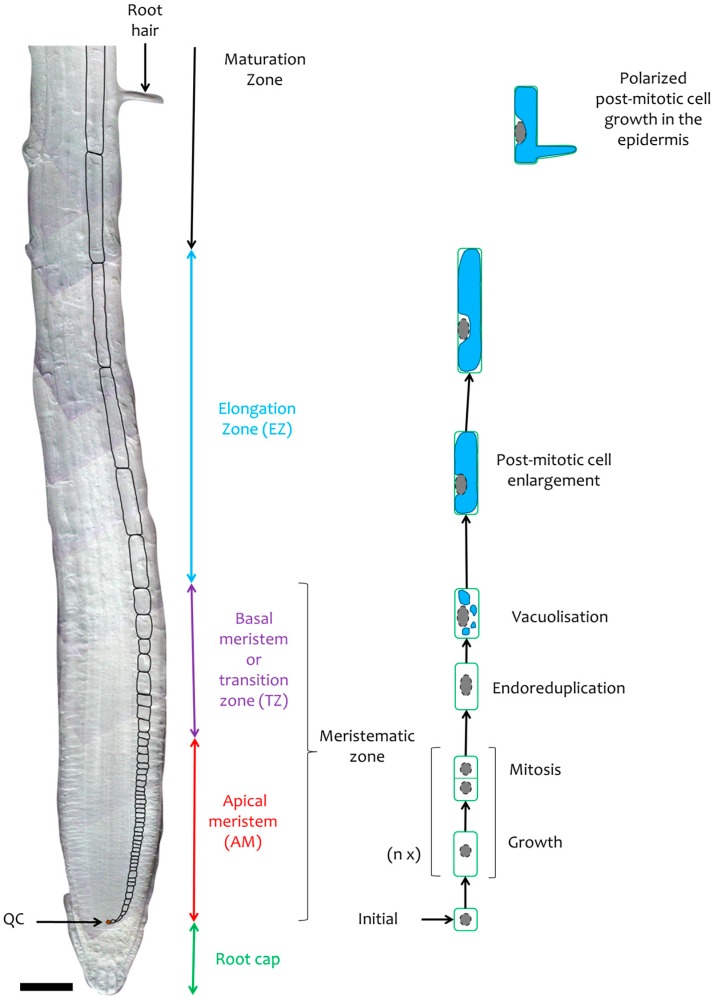
The cellular mechanims involved in primary root zonation. Photomontage of differential interference contrast microscopy pictures of the *A. thaliana* primary root. Bar 50 µm. The black contours of cortical cells highlight the increase in cell size. Within the apical meristem new cells are formed by successive division (n x: non-initial cells divide n times in the meristem), each preceded by an increase in size corresponding to cell growth. In the basal meristem most cells have stopped dividing and undergo endoreduplication and vacuolization at the initiation of cell expansion. In the elongation zone all cells have stopped dividing and expand rapidly. In the maturation zone, cells have reached their final length and some specialized epidermal cells go through polarized cell enlargement leading to the formation of root hairs.

The longitudinal axis of the root can be viewed as a developmental timeline (Figure 2). Younger cells that originate from the initials go through a proliferation phase in the apical part of the meristematic zone (Figure 2). In the basal meristem, generally only a few cells can still divide while the large majority stop dividing and progressively elongate. This region of the root, also called the transition zone, is frequently located at the position where cortex cells start to clearly enlarge. In the elongation zone, cell size can increase up to 20 times by rapid vacuolar expansion [9]. Cells reach their final length and undergo ultimate differentiation in the maturation zone. This zone is typically characterized by the outgrowth of root hairs in specialized cell lines of the epidermis. In *A. thaliana*, divisions rarely occur in the transition zone. The transition from the dense proliferating cells of the meristem to the more translucent and vacuolised cells of the elongation zone is clearly visible in flowering plants (Figure 2).

### 2.2. Cell Proliferation

#### 2.2.1. Essential Cell Cycle Regulations in Plants

Since all cells that expand in the elongation zone are produced by proliferation in the meristem, the control of the cell cycle is important for root growth and morphology. The cell cycle progression through the G1, S, G2 and M phases is regulated by cyclin and cyclin-dependent kinase (CDK) complexes, which phosphorylate key target proteins [10,11]. CDKs are activated by three classes of cyclins, A-type (CYCA), B-type (CYCB) and D-type (CYCD), and inhibited by two small families of proteins, p27^KIP1^ related proteins (KRPs) and the plant specific SIAMESE (SIM) and SIM-related (SMR) proteins. Cell cycle phase specific regulation of CDK activity is controlled by the striclty ordered synthesis and degradation of cyclins and CDK inhibitors. Degradation is achieved by the ubiquitin proteasome pathway through the F-box selective targeting of cyclins and CDK inhibitors by the Skp1/Cullin/F-box (SCF)-related complex at G1/S and the anaphase-promoting complex/cyclosome (APC/C) at G2/M.

The initiation of the cell cycle is controlled by a general G1/S phase module comprising the plant homologues of the animal transcription factor E2F which are sequestered by the plant homologue of the animal transcriptional repressor RETINOBLASTOMA (RB)-related (RBR). CYCD/CDKA;1 complexes control the G1/S transition by phosphorylating RBR, thereby liberating the E2F homologues and their associated DIMERISATION PARTNERS (DPs) which go on to promote the transcription of S-phase genes [12,13,14].

Genes involved in progression to G2/M phase, contain an M-phase-specific activator (MSA) element in their promoter region, but a clear-cut role for cis-elements and transcription activators or repressors in cell cycle gene expression is not yet clearly delineated [15,16,17,18,19]. Recent data shows that the E2F-DP-RBR pathway is also active at the G2/M transition [20]. While CYC/CDK complexes are decisive actors of cell cycle progression, other kinases also play important roles including the DNA integrity checkpoint Wee1, the energy depletion sensor Sucrose non-fermenting-1-Related protein Kinase 1 (SnRK1), and the plant orthologue of the mammalian adenosine monophosphate-activated protein kinase (AMPK) [21,22,23]. Environmental cues and organ functional specificity will also add variations to the networking of the cell cycle interactome [24,25].

#### 2.2.2. Quiescent Center and First Asymmetric Divisions

The QC is established during embryogenesis and its cells remain at G1 in an undifferentiated state while suppressing division in the surrounding initials (Figure 1).

**Figure 3:**
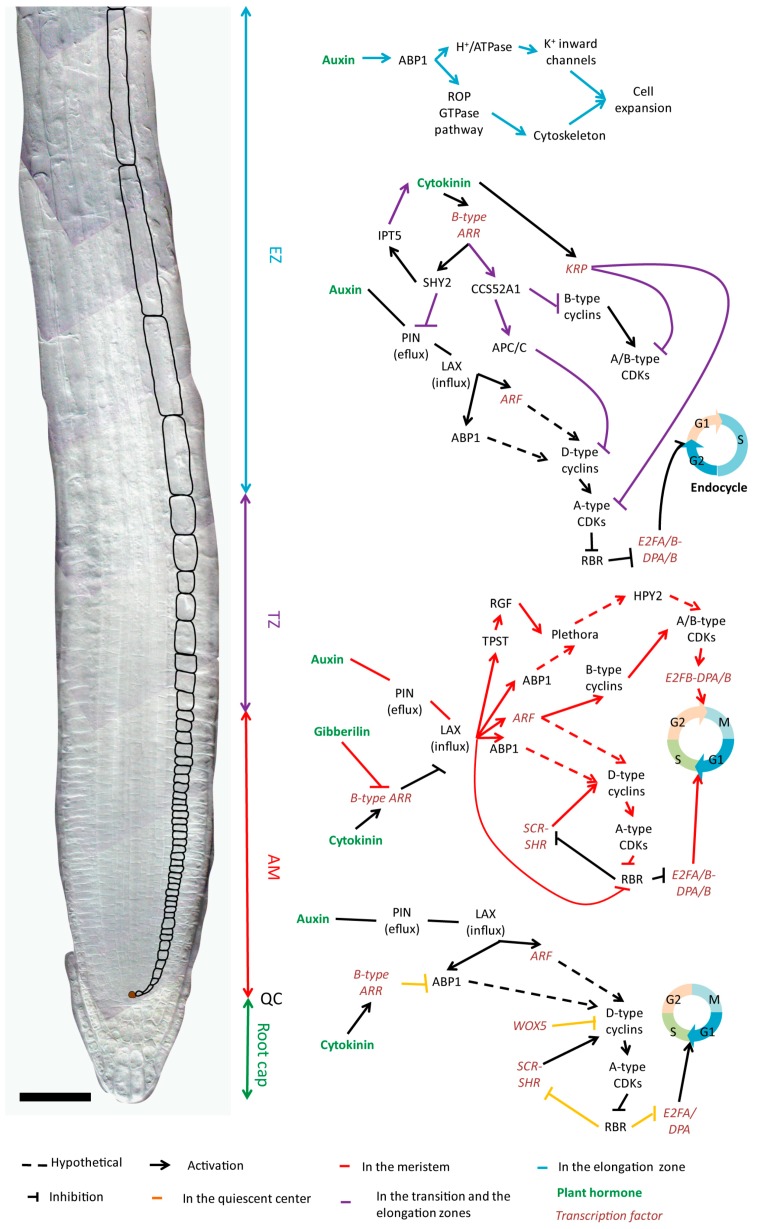
Proposed model of the complex interplay between hormones and cellular growth regulators during root growth. In each zone of the root, hormones induce specific regulatory processes that influence cell cycle progression or cell expansion (see main text for details). Bar 50 µm.

In roots, RBR is necessary for restricting the amount of initials around the QC, but it does not affect cell cycle duration [26]. In QC cells and initials, transcriptional regulation controls the expression of CYCDs which activate CDKs, and results in the repression of RBR through phosphorylation. As RBR is expressed throughout the root, the expression pattern of the cyclins will, therefore, determine cell cycling in conjunction with tissue-specific identity factors [26,27]. For example, CYCD3;3 is expressed in dividing initials of the columella, the epidermis, the lateral root cap and the stele but is not expressed in the QC due to the repressor activity of the QC specific transcription factor WUSCHEL-RELATED HOMEOBOX 5 (WOX5) [28,29]. Therefore, division is repressed in the QC because RBR is not phosphorylated and can sequester factors including cell cycle specific E2Fs and developmental transcription factors such as SCARECROW (SCR). This prevents the dimerization of SCR with the mobile stele-specific transcription factor SHORTROOT (SHR) that can promote asymmetric division through activation of specific CYCD transcription (Figure 3).

#### 2.2.3. Apical Meristem

Once cells have committed asymmetric division, they will actively divide several times. These processes are achieved by the stoichiometry of RBR-free and RBR-bound E2Fs, particularly S phase specific E2FA [13,30]. After DNA replication at S phase, CYCBs associate with CDKA or CDKB to promote G2/M transition [31], CYCB1;1 being a typical division marker of the apical meristem [32]. In a manner analogous to E2F in G1/S, the G2 CYC-CDKA/B complexes phosphorylate several G2-specific transcription factors. The CYCB/CDK complex regulates the Kinesin-like protein NACK1, which activates, after the metaphase, a MAPK cascade leading to cell plate formation and cytokinesis [33,34]. As each division is being preceded by an increase in cell content, an increase in ribosomal activity and protein synthesis is required.

#### 2.2.4. Basal Meristem

As cells reach the transition zone, their proliferation slows down and their size begins to increase. At this point cells are clearly marked by CCS52A, an activator of the APC/C complex [35]. Cells often enter an alternative cell cycle known as the endocycle which is characterized by DNA endoreduplication. At a molecular level, this shift from a mitotic cycle to an endocycle may be triggered by a reduction in CDK activity to a level that exceeds its threshold for DNA replication but stays below its threshold for mitosis [12].

CDK inhibitors, such as KRPs [12,36] and SIMs/SMRs [37], regulate the transition from mitosis to endocycle. Indeed, KRP5 has been shown to positively regulate root cell enlargement and endoreduplication [38]. Inhibition of the M-phase during the endocycle may also be caused by premature activation of the FIZZY-RELATED (FRZ) protein, which directs the APC/C to degrade mitotic cyclins and promote exit from M-phase [39,40]. In most *A. thaliana* epidermis cells [41] and in collet hair cells [42], a positive correlation between DNA content, nuclear volume and cell volume has been reported.

### 2.3. Cell Expansion, a Major Contributor to Root Growth

#### 2.3.1. Post-Mitotic Cell Enlargement

In the elongation zone, cells stop dividing and cell growth occurs exclusively through rapid anisotropical cell expansion. Anisotropical cell expansion requires an increase in turgor pressure that occurs through the uptake of water into the vacuole [43] and an irreversible extension of the cell wall through wall loosening and the deposition of new material.

The cell wall is made of cellulose microfibrils connected through hemicellulose and embedded in a pectin matrix composed of polysaccharides. The composition of this polysaccharide network changes along the root, with a decrease in pectin and an increase in the hemicellulose xylan, from the meristematic zone to the elongation zone and the maturation zone [44]. Cell wall loosening results from modifications of molecular interactions within the cell-wall network [45]. For example, extracellular acidification activates expansins, which are hypothesized to break hydrogen bonds between hemicelluloses and cellulose microfibrils, thus facilitating hemicellulose sliding along the cellulose scaffold [46].

Several membrane proteins are also important for cell expansion, including the cellulose synthases (CeSAs) [47]. Furthermore, uptake of K^+^ in the vacuole is likely to contribute to the osmotic force driving the water uptake necessary to sustain expansion [48,49]. The NADPH oxidase RHD2 produces reactive oxygen species (ROS) that cause hyperpolarization of the membrane and activation of Ca^2+^ channels resulting in an uptake of Ca^2+^, which is also required for cell elongation [50,51].

The cytoskeleton and particularly the transverse orientation of cortical microtubules control the polar expansion of cells by providing a track for cellulose synthase to deposit new cellulose microfibrils. The microtubule arrays may influence the orientation of the cell-wall microfibrils and consequently the direction in which the cell wall can be stretched during the next cycle of cell wall extension [52,53]. Furthermore, cell expansion driven by osmotic force requires synthesis of membrane lipids and cell wall components. How these new components are integrated is determined by the cytoskeleton, which thereby acts as a major determinant of growth orientation [54].

#### 2.3.2. Polarized Root Hair Growth

The elongation process gradually stops in the maturation zone mainly due to a decrease in cell wall extensibility [55]. However, root cells reach not only their final size but also their final form in the maturation zone which can be associated with particular growth processes. In the *A. thaliana* root epidermis, non-hair cell files alternate with root hair cell files. This epidermal cell patterning is the result of positional signal perception and a subsequent gene expression cascade [56,57] (Figure 2). Root hair growth is similar to common cell elongation but the main difference is the polarized growth involving a ROS-mediated, tip-focused Ca^2+^ gradient maintained by the microtubules [56]. Moreover, actin microfibrils contribute to polarized growth by playing a role in the transport of secretory vesicles that contain cell wall and membrane components from the Golgi complex to the root hair tip.

### 2.4. A Cross-Talk between Plant Hormones Controls the Balance between Cell Division and Differentiation

Root zonation is the result of a spatial separation between proliferation and elongation. These two cellular processes are coordinated by systemic signals, mainly plant hormones. Simulations using a model based on the antagonistic role of auxin and cytokinin [58] reproduced visual and kinematic observations, such as the expected increase and decrease in the size of the meristem upon addition of auxin and cytokinin [59]. This suggests that a cross talk between these two hormones is essential for root morphogenesis and growth.

#### 2.4.1. Establishment of a Hormone Gradient along the Root

**Figure 4:**
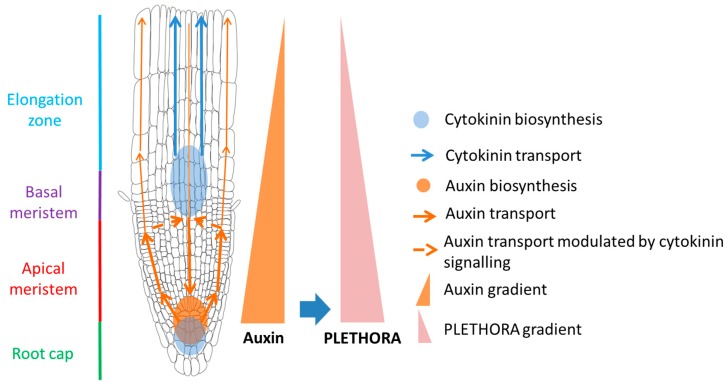
Schematic model of hormonal transport and auxin and PLETHORA gradients in the root (see main text for details).

In root tips, the *YUCCA* (*YUC*) genes which encode the rate limiting enzymes for auxin biosynthesis are strongly expressed in the columella, the QC and the surrounding initials [60]. Isopentenyltranseferase 5 (IPT5), which is involved in cytokinin biosynthesis is highly expressed in the columella and the vascular tissues of the transition zone suggesting that cytokinins are synthetized in these areas [61] (Figure 4).

Auxin is transported throughout the plant via specialized carriers on the cell membranes, the PIN-FORMED family of auxin efflux carriers [62] and the AUXIN1/LIKE-AUX1 (AUX/LAX) family of influx carriers. Within the vascular tissues of the root, auxin moves towards root apex via PIN efflux carriers like PIN1 [63,64]. Inside the lateral root cap and epidermal cells, PIN2 and AUX1 create a shootward auxin flux [62,65]. In the transition zone, where cytokinin is synthesized, the B-type cytokinin response transcription factors *ARABIDOPSIS* RESPONSE REGULATOR (ARR) 1 and ARR12 directly promote the expression of SHY2/ Indol Acetic Acid 3 (IAA3). The latter binds to and inhibits auxin response transcription factors AUXIN RESPONSE FACTORS (ARFs) to repress auxin signaling, including *PIN* transcription [61]. Therefore, SHY2 prevents an increase in auxin levels in cells at the transition zone and favors auxin accumulation in the apical meristem by causing a reorientation of the auxin flow towards the root tip [61,66] (Figure 4). In the apical meristem, high auxin levels promote SHY2 ubiquitination via the SCF^TIR1/AFB^ complex and its subsequent degradation by the 26S proteasome, thus sustaining PIN activity [67]. Therefore, local auxin biosynthesis combined with auxin transport and its repression by cytokinin are involved in forming the auxin gradient within the root (Figure 4).

The auxin distribution along the root resembles that of *PLETHORA* (*PLT*) expression. The *PLT* genes encode APETALA 2 domain transcription factors that control different aspects of root development in a dose dependant manner [68]. High auxin levels generate high PLT concentrations within a narrow zone of the root meristem where *PLT* transcription occurs. This auxin-mediated induction of *PLT* depends on the sulfonation of small peptides called ROOT GROWTH FACTORS (RGF), by a tyrosylprotein sulfonyltransferase. Indeed the *RGF1*, *2* and *3* triple mutant displays a shorter meristem and a reduced *PLT2* expression zone [69]. A gradient of PLT proteins is subsequently generated through slow growth dilution and cell-to-cell movement along the longitudinal root axis [68].

#### 2.4.2. Auxin and Cytokinin Regulate Proliferation and Elongation

In the QC, auxin influx is repressed by cytokinin signaling. In fact, ARR1 represses *LAX2* transcription by binding to the regulatory region of this gene [70]. In dividing initials, down-regulation of ARR1 expression by gibberellin, another phytohormone, ends LAX2 inhibition and allows auxin uptake [66]. Auxin, as a mitogen factor, plays at specific cellular concentrations a crucial role during the division of initial cells. Indeed, primary roots treated with exogenous auxin and the inhibitor of polar auxin transport N-1-Naphthylphthalamic acid have enhanced auxin accumulation at the root tip, which leads to increased CYCD6;1 expression in the cortex/endodermal initials and endodermal cells [71].

High auxin levels induce the expression of CYCs and CDKs like CDKA;1 in *A. thaliana* seedlings [72]. The permissive effect of auxin on cell division is dependent on the receptor AUXIN BINDING PROTEIN 1 (ABP1), which is essential for the maintenance of cell division in the meristematic tissues at different levels. ABP1 acts on the CYCD/RBR pathway regulating the G1/S transition and affects the PLT gradient [73]. Although their function has not been confirmed, auxin responsive elements (AuxREs) are found in the promoter regions of cyclins, such as CYCA2 [74] or CYCB1;1 [18], which are essential for G2-M phase transition and thus for cell proliferation. CDKB expression in the apical meristem is regulated by an E3 SUMO protein ligase called HIGH PLOIDY2 (HPY2), which functions downstream of the PLT transcription factors [75]. The reduction of CDKB proteins and to a lesser extent of its transcripts in *hpy2* (high ploidy 2) mutants suggests that HPY2-mediated sumoylation of CDKB2;1 is necessary for its accumulation, and, therefore, for endocycle repression and meristem maintenance [75,76]. Thus, high levels of auxin in the meristem might cause increased CDKB expression through PLETHORA and HPY2 activation. All this shows that auxin plays a major role in the initiation and the maintenance of mitotic activity in the meristem.

In the transition zone and the elongation zone, CCS52A1, an activator of the E3 ubiquitin ligase APC/C that promotes mitotic cyclin degradation is expressed [35,39]. CCS52A1 is also up-regulated by the B-type cytokinin response regulator ARR2 [77]. Cytokinin, therefore, plays a role in determining the root meristem size by repressing auxin signaling and enhancing the degradation of mitotic regulators by the APC cyclosome at the transition zone thereby promoting endoreduplication. A functionally redundant protein, CCS52A2 is expressed in the columella suggesting a similar repression of cell division at the QC by cytokinin [35].

Auxin levels also drive cellular expansion in the elongation zone by stimulating cell wall loosening and water uptake. Upon binding with auxin ABP1 activates the H^+^**-**ATPases responsible for cell wall acidification and the potassium channels causing K^+^ uptake [78,79]. However, auxin has also been shown to affect cytoskeleton organization. Indeed, auxin treatment of maize roots affects microtubule orientation [80]. Moreover, auxin seems to be implicated in the reorganization of actin filaments into fine cortical strands. Recent studies show a complex regulatory loop between auxin and cytoskeleton reorganization involving ADP-ribosylation factors and the ROP GTPase signaling pathway [81], and leading in presence of high auxin concentrations to cell expansion inhibition [82].

In summary, cells undergo different stages as they progress through the primary root. At first, initials start dividing as E2F repression by RBR is alleviated. In the apical meristem, cell proliferation is accelerated thanks to an increase in CDK activity. As the latter decreases due to cyclin degradation and CDK inhibition, cells enter the endocycle which is required for cell elongation involving cell wall expansion and a rise in osmotic force. Cells reach their final size and form in the maturation zone, where some specific epidermal cells develop root hairs through polarized cell expansion. However, systemic signals, mainly auxin and cytokinin, are necessary for longitudinal zonation during root growth by regulating the transition from cell proliferation to elongation. The fact that auxin is synthesized from the amino acid tryptophan [83,84] and cytokinin from ATP and ADP [85] suggests that the mechanisms regulated by these hormones are linked to amino acid and nucleotide metabolism, which in turn are related to environmental factors such as nutrient availability. In animals and in yeast, the highly conserved eukaryotic target of rapamycin (TOR) kinase has been shown to connect nutrient and hormonal signaling with growth. A similar interaction might also exist in plants.

## 3. The TOR Signaling Pathway, a Master Regulator of Root Growth Adaptation to Nutritional Conditions

In this section we will first summarize the TOR pathway in mammals and in yeast, and then proceed with the current knowledge on TOR in plants. The role of TOR in proliferation and cell expansion in roots will be presented before opening to more general aspects of plant TOR signaling based on evidence that has not necessarly been demonstrated in roots but offer interesting leads for understanding root growth regulation.

### 3.1. The TOR Pathway a Conserved Major Regulator of Cell Growth in Eukaryotes

#### 3.1.1. The TOR Pathway in Yeast and Animals

Target of rapamycin (TOR) is a large and highly conserved Ser/Thr kinase belonging to the family of phosphatidylinositol 3-kinase-related kinases (PIKKs). It was discovered in the budding yeast *Saccharomyces cerevisae* through a genetic screen for mutants resistant to rapamycin, an immunosuppressant that blocks human T cell activation and proliferation [86]. This protein is the central component of the TOR signaling pathway, which regulates cell growth and metabolism in response to environmental cues in eukaryotes. Many components of this pathway have been studied in human cells and animals as they are involved in cancer and metabolic diseases. In both mammals and yeast, the TOR kinase exists within two distinct multi-protein complexes, TORC1 and TORC2, that regulate different molecular mechanisms required for cell and organism growth [87]. The mammalian TORC1 consists of TOR, Regulatory Associated Protein of mTOR (RAPTOR) and the Lethal with SEC13 protein 8 (LST8). The allosteric inhibitor rapamycin can inhibit TORC1 through the formation of a ternary complex between TOR and the peptidyl-prolyl cis-trans isomerase FKBP12 (FK506 and rapamycin-Binding Protein of 12 kDa). When active, yeast and mammalian TORC1 positively regulate protein synthesis, cell-cycle progression, and energy metabolism, while inhibiting stress responses, such as autophagy. The best characterized targets of mammalian TORC1 are S6 kinase 1 (S6K1) and the eukaryotic initiation factor 4E-binding protein (4E-BP1). 4E-BP1 sequesters eukaryotic Initiation Factor 4E (eIF4E), which is involved in the initiation of cap-dependent translation of specific mRNAs encoding proteins involved in cell growth, proliferation, and survival. Alterations in translation control via 4E-BP1 are considered as important steps in the transformation of healthy cells into tumor cells [88]. S6K1 phosphorylates the ribosomal protein S6 (RPS6) thereby connecting the TOR pathway with ribosomes. mTORC1 also regulates ribosome biosynthesis and translation through RNA polymerases I and III which are responsible for rRNA and tRNA synthesis. The mammalian TORC2, which consists of TOR, Rapamycin-Insensitive Companion of mTOR (RICTOR), LST8 and stress-activated map kinase interacting protein 1 (SIN), is insensitive to rapamycin [89]. The main known function of TORC2 in yeast and mammals is to regulate spatial control of cell growth via the actin cytoskeleton. Both TORC1 and TORC2 activity are modulated in response to environmental factors. For instance in yeast, TORC1 is activated by nutrients through interactions with the vacuole, and inhibition of TORC1 mimics carbon, nitrogen, phosphate or amino acid starvation. Other stresses such as high salt, oxidative stress, and high temperature, are also sensed by TORC1 and TORC2. TORC1 is also inhibited by AMPK, a sensor of energy homeostasis [90]. Moreover, TOR integrates systemic signaling in animals as both TOR complexes are activated by insulin-like growth factors via phosphatidylinositol 3-kinase (PI3K) and Phosphatase and TENsin homologue (PTEN) [87].

#### 3.1.2. Conservation of the TOR Pathway in Plants

Using complete genome sequences, Serfontein and coworkers showed that the current TOR pathway was built up from a simpler one present in the ancestral eukaryote. This ancient pathway contained PTEN, TOR, RAPTOR, AMPK, LST8, PI3K and S6K, which are all present in plants [91,92]. Additional elements were added to the pathway during evolution. Several members of the viridiplantae lineage including plants, red algae, and green algae are not, or hardly, sensitive to rapamycin [93,94,95,96]. However, this is not due to the absence of a TORC1 complex but rather due to the fact that the corresponding FKBPs do not carry the amino acids that are critical for the interaction between FKBP and rapamycin in animals and yeast. No 4E-BP homologues are found in plants, suggesting that either plant TORC1 does not regulate cap-dependent translation or that it does, but through a mechanism different from that of yeast and mammals. Finally, there is no evidence of a plant TORC2 as no homologues of RICTOR and SIN1 are found in the genome of *A. thaliana* [92]. However, the possibility of existence of other TOR complexes in plants is an interesting lead for future studies.

### 3.2. A Key Regulator of Plant Cell Growth

#### 3.2.1. TOR Is a Global Regulator of Plant Growth

Analysis of *A. thaliana* plants carrying a translational fusion with the *GUS* reporter gene shows that TOR is expressed in embryos and all meristems, including primary and lateral roots meristems, as well as shoot apical and floral meristems [97]. *TOR* loss of function mutants stop growing at an early stage of embryo development (16 to 32 cells), indicating that TOR is essential for plant growth [97,98]. Furthermore, inducible silencing of *TOR* impairs post-embryonic growth, whereas plants slightly overexpressing *TOR* are bigger than wild type plants [99,100,101]. Plant growth is also reduced in mutants for components of the TORC1 complex, such as RAPTOR and LST8 [102,103,104], whereas overexpression of the PP2A regulatory subunit Tap46, a conserved mediator of TORC1 signaling enhances plant growth [105]. Although most of these observations were not focused on roots, they highlight the role of plant TORC1 as a major regulator of plant growth.

#### 3.2.2. TOR Function in the Root Meristem

**Figure 5:**
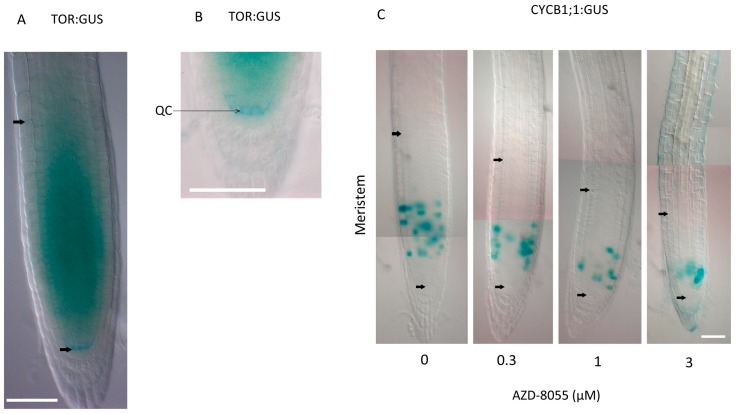
TOR is expressed in the meristem and the QC and regulates meristem size and the zone of CYCB1;1 expression in the apical meristem. Differential interference contrast microscopy pictures of GUS stained *A. thaliana* primary roots showing the meristem, delimitated by black arrows. (**A**,**B**) Expression of TOR:GUS in roots of 4 days-old plants stained for 4 h [97]; (**B**) Evidence of TOR:GUS expression in the quiescent center; and (**C**) Effect of asTORis on CYCB1;1*-*GUS expression in *A. thaliana* roots. Pictures were taken 2 days after transfer of 3 days-old plants onto medium containing the indicated concentrations of asTORi (µM) (from Montané and Menand, 2013 [1]). Bars, 50 µm (**A**–**C**).

In primary roots, the TOR::GUS translational fusion protein is expressed in the QC, the apical meristem and the basal meristem (Figure 5A,B). The expression of TOR in the QC suggests that its presence is not sufficient for the induction of cell division. The TOR signaling activity capable of promoting growth and cell division may, therefore, be inhibited in the QC due to the absence of essential partners, targets or inputs. The concomitant expression of TOR in both the apical and basal meristems suggests a role for TOR in both proliferation and cell expansion in the root. Indeed, several studies showed the importance of TOR during primary root growth [1,2,3]. For example, treatment of *A. thaliana* roots with recently developed ATP competitive TOR inhibitors (active site TOR inhibitors or asTORis) results in a rapid inhibition of root growth in a *TOR* gene-dose-dependent manner [1]. AsTORis reduce meristem size in a dose-dependent manner through reduction of the number of cells in the meristematic zone, which is concomitant with earlier cell differentiation. This suggests a role for TOR in regulating the proliferation capacity of cells. Indeed, treatment with asTORis reduces the number of cells expressing the CYCB1;1-GUS marker, indicating that there is a reduction in the number of dividing cells in the apical meristem (Figure 5C). However, the density of the GUS staining in the marked cells did not change, suggesting that asTORis do not affect the G2 phase when CYCB1;1 is normally expressed [1]. Xiong and coworkers showed that in inducible *TOR* RNAi lines, TOR inhibition leads to a decrease in DNA synthesis and in transcription of E2FA target genes in root meristems [3]. Both studies indicate that TOR seems to be involved in the progression through the G1 to S phase. Furthermore, *in vitro* kinase assays revealed that E2FA is directly phosphorylated by *A. thaliana* TOR (AtTOR) even after removal of the RBR binding domain, suggesting that E2FA can be regulated by AtTOR independently of the CYC-CDK-RBR pathway [3]. The reduction in root length, in root meristem size, and in CYCB1;1-GUS marked apical meristem size by asTORis occur together, showing a highly coordinated process where cell division and growth are slowed down [1]. The reduction in whole plant size by asTORis suggests that the inhibition of shoot, hypocotyl, and root growth is also coordinated. This suggests a downstream position of TOR with respect to overall developmental patterning. Indeed, plants treated with asTORis are also not affected in the patterning of epidermal hair cells and non-hair cell files [1]. Moreover, in *TOR* RNAi or rapamycin treated roots, the expression pattern of the transcription factors WOX5 in the QC and PLT1 in the basal meristem is unchanged [3]. This suggests that TOR does not regulate quiescent cell maintenance and initial cell establishment, and is in accordance with the idea that TOR regulates proliferation independently or downstream of developmental patterning. Finally, reduced cell and root hair sizes in the mature zone of asTORis treated plants suggest that TOR also plays a role during cell expansion. AsTORis also inhibit the growth of roots and root hairs in the monocots rice and millet and the dicots *Lotus japonicus* and *Nicotiana benthamiana*, indicating that the regulation of root growth by TOR is conserved in angiosperms [1].

#### 3.2.3. Regulation of Cell Expansion by TOR

TOR:GUS is principally observed in the apical meristem, which, at first, suggested that TOR is not involved in cell expansion [106]. However, some TOR:GUS staining can also be seen in the whole root meristem, including the basal meristem where expansion occurs (Figure 5A). Furthermore, as discussed earlier, cells expansion leads to a large increase in cell size that could simply lead to a dilution of the GUS signal (and also the TOR protein) along the longitudinal axis. Indeed, after long staining of TOR-GUS plants (24-h incubation in GUS staining solution), slight GUS staining can also be observed in the elongation zone (Montané and Menand, unpublished), suggesting a function for TOR in cell expansion. Functional studies are in agreement with this hypothesis. As mentioned above, asTORis treated plants have smaller cells and root hairs in the mature zone, suggesting a role of AtTOR in the regulation of cell expansion [1]. In roots of plants expressing yeast FKBP12 and treated with rapamycin, changes in pectin polysaccharides**-**associated proteins are observed, suggesting that TOR might play a role in cell wall formation [107]. In addition, in rapamycin treated yeast FKBP12 lines a reduced root hair ROS-dependant signal is observed. ROS accumulation is essential for the elongation of developing root hairs, which may offer some explanation as to why TOR inhibited plants have shorter root hairs [2]. Furthermore, transcriptome analysis on rapamycin treated plants expressing yeast FKBP12 and on TOR RNAi lines showed down regulation of genes involved in cell wall formation, such as expansins and extensins [2,104,108]. Therefore, in addition to controling cell proliferation, AtTOR could control cell expansion in the elongation zone and also polarized cell growth. However, the role of TOR in endoreduplication in the basal meristem remains to be investigated.

#### 3.2.4. S6K, an Important Element of the Plant TOR Pathway.

Experiments on protoplasts and young seedlings have shown that *A. thaliana* S6K1 (AtS6K1) phosphorylation at the conserved T449 site is inhibited by addition of an asTORi or by induction of *TOR* silencing, indicating that S6K1 phosphorylation is TOR dependent [101,109,110]. Indeed, AtS6K1 was shown to interact with RAPTOR when overexpressed in tobacco leaves, indicating a physical link between AtS6K1 and TORC1 [95]. AtS6K2 expressed in mammalian cells is able to phosphorylate the ribosomal protein S6, suggesting a conservation of this activity [111]. Furthermore, in *A. thaliana* cell cultures, S6K dependent S6 phosphorylation is induced in stationary cells that are given fresh media, and this activation of S6 phosphorylation requires both auxin and cytokinin [112]. There is no functional analysis of S6Ks in roots, but S6 was studied in both maize and *A. thaliana* roots. Nine phosphorylated S6 isoforms were found in ribosomes extracts from maize root tips [113]. Anoxia and heat shock were shown to reduce the levels of the most highly phosphorylated forms of S6. High S6 phosphorylation was also inhibited in both *A. thaliana* cell cultures and root tips by LY294002 [112,113] a broad-spectrum ATP-competitive inhibitor of PI3K and TOR. PI3K acts upstream of TOR in animals, but can also inhibit other kinases [114]. Although LY294002 is not very selective, this suggests that S6 phosphorylation is regulated via the TOR pathway [87]. Notably, the inhibition of the plant TOR pathway by LY294002 is supported by the report that LY294002 generates a root phenotype similar to asTORis [1]. Finally, *A. thaliana* mutants in genes encoding RPS6, the target of S6K, show a reduction in root growth and meristem size, similar to that observed after TOR inhibition [2,115]. Together, these data support the hypothesis of an S6K activity that is dependent on TOR and that could regulate ribosome activity via S6 phosphorylation in the root meristem.

As in mammals, plant S6Ks seem to have other targets than RPS6 [116]. In *A. thaliana* protoplasts, downregulation of S6K1 and S6K2 through transient RNA interference leads to increased levels of E2FB [117]. Indeed, immunoprecipitation assays showed an interaction between S6K1 and RBR, and this interaction is required for the nuclear localization of RBR, as well as the E2F-dependent expression of cell cycle genes [117]. Transcriptomic data indicates that AtS6K1 and AtS6K2 are predominantly expressed in the transition and elongation zones of the primary root respectively [117,118]. Therefore, although not demonstrated in roots, we speculate that S6Ks might similarly be negative regulators of proliferation via RBR and E2FB in the root transition and elongation zones. In S6K1 RNAi lines, and in S6K1 and S6K2 double mutants, enhanced ploidy levels were also observed in leaves and flowers, suggesting that S6K might be one of the links between TOR and endoreduplication in the basal meristem.

#### 3.2.5. Regulation of Plant Metabolism by TOR

Transcriptomics and metabolomics of artificial microRNA (amiR) lines of *TOR* (amiR-TOR) showed that TOR regulates major metabolic pathways. Indeed, most biological processes downregulated by *TOR* silencing were anabolic activities, such as amino acid and nucleotide synthesis [119]. On the contrary, catabolic activities were generally up-regulated, as well as lipid metabolism, reflecting a deep reorientation of cell metabolic activity. *A. thaliana* *tor* mutants showed reduced expression of 5S, 18S, and 25S rRNAs in embryos, whereas overexpression of TOR or its kinase domain alone lead to elevated rRNA expression [98]. In accordance with this result, a more recent study proposed a model in which the TOR pathway mediated phosphorylation of RPS6 prevents formation of the RPS6-HD2B-NAP1 complex (Ribosomal Protein S6-Histone Deacetylase 2B-Nucleosome Assembly Protein 1), which represses rRNA transcription [120]. This shows that TOR regulates rRNA expression, which is essential for ribosome assembly and translation. Protein translation is initiated with the recruitment of ribosomes to mRNAs to form polysomes. Partial silencing of *TOR* in ethanol induced *TOR* RNAi lines, as well as rapamycin treatment of plants expressing yeast FKBP12, result in the reduced accumulation of polysomes [96,100]. Therefore, TOR might regulate protein synthesis via both the assembly of ribosomes, as well as the formation of polysomes. Moreover, TOR has also been linked to autophagy, which is a protein degradation process by which cells recycle cytoplasmic contents under stress conditions or during senescence. Numerous *AUTOPHAGY (ATG)* genes have been identified in plants. In *TOR* RNAi lines, expression of *ATG* genes was increased, especially that of *ATG18a*, and autophagosomes formed in root apical meristem cells [108]. TOR, therefore, seems to be a negative regulator of autophagy in plants, as in animal and yeast. In estradiol-induced amiR-TOR plants starch and triacylglycerol (TAG) accumulation was observed [119]. As starch and TAGs are important forms of carbon storage in plants, this suggests that the TOR pathway might redirect the carbon flow in accordance with the needs of the plant. Altogether, this evidence indicates that plant TOR promotes growth by regulating anabolism and protein synthesis, as well as carbohydrate distribution and nutrient recycling. Beyond that, active TOR may adapt its control of these processes to different cellular states modes, such as proliferation or elongation, and depending on environmental factors like nutrient availability or abiotic stresses.

### 3.3. TOR as an Integrator of Environmental and Hormonal Signaling

#### 3.3.1. Integration of Nutrient Status by TOR

Carbon, either extracted from the atmosphere through photosynthesis, or from the soil, as well as nitrogen, mainly sourced from soil nitrate, are essential inputs for plant metabolism. Along with other nutrients, they are critical for nucleic acid, amino acid, lipid, and sugar biosynthesis, which form the main building blocks necessary for plant growth.

A recent study showed that *A. thaliana* seedlings germinated in photosynthesis constrained and sugar-free liquid medium initiated light-mediated development, but entered a mitotic quiescent state with arrested root meristem activity and growth after depletion of endogenous glucose at three days after germination [3]. Photosynthesis activated by higher light and ambient CO_2_ or addition of glucose was sufficient to promote rapid root growth. In estradiol-inducible *TOR* RNAi lines and rapamycin treated plants, light or glucose driven reactivation of root growth was inhibited. This indicates that shoot photosynthetic-derived glucose drives TOR signaling, and leads to root meristem activation. The use of the glycolysis blocker 2-deoxyglucose (2-DG) and a mitochondrial electron transport inhibitor antimycin A (AMA) showed that glucose metabolism via glycolysis-mitochondrial energy relays is required for TOR-dependent root meristem activation [3]. In another study, the response of yeast FKBP12 expressing plants to growth activation by different nutrients was analyzed [2]. Growth activation either by an increase in nitrogen or sucrose concentration or light intensity are inhibited by rapamycin in a similar manner. This suggests the existence of a global nutrient-TOR pathway that adapts plant growth to nutrient availability.

#### 3.3.2. Potential Connection with AMPK

In yeast, the TOR pathway has been shown to adjust growth and metabolism to available resources, nutrient and energy levels inside and outside the cell [121]. Sucrose non-fermenting-1 (SNF1) is a Ser/Thr energy sensing kinase involved in the adaptation to glucose deprivation in yeast, and allows the utilization of alternative carbon sources. Orthologues of SNF1 have been found in all eukaryotes: AMPK in mammals and SnRK1 in plants [122,123]. In mammals, AMPK, which is activated by the low energy signal AMP, has been shown to phosphorylate an inhibitory and conserved Ser residue in the RAPTOR protein, allowing direct control of TORC1 activity [90]. Although this has not yet been shown in plants, conservation of the phosphorylation site responsible for TORC1 repression on plant RAPTOR suggests the existence of a similar mechanism [124]. However, a recent study has shown that *A. thaliana* SnRK1 is different from AMPK and SNF1 in terms of subunit composition, and is insensitive to AMP [125]. However, this does not exclude the possibility that a plant specific SnRK1/TOR regulatory module might connect nutrient sensing to the cell growth machinery, although with a signaling mechanism that would be drastically different from that of mammals and yeast.

#### 3.3.3. TOR and Auxin

In *TOR* RNAi induced lines or rapamycin-treated plants, the distribution and the activation of auxin and cytokinin signaling reporters: DR5::GFP and TCS::GFP, were not affected in the primary root [3]. This suggests that the TOR pathway does not control the hormonal transport and accumulation that are essential for maintaining root zonation during growth. However, induction of amiR-TOR leads to the accumulation of transcripts encoding the peptides RGF6 and RGF9 [3], which are homologues of the RGF1, RGF2, and RGF3 peptides that regulate PLT2 expression, but have a more diffused expression pattern in the root meristem [126]. Therefore, TOR could potentially mediate auxin signals and for example could induce CDKB expression via RGF6 and RGF9, which trigger PLT gene expression in the meristem. Thus, the TOR pathway may act downstream of phytohormones in order to regulate cell cycle progression in the meristem. Indeed, exogenous addition of the auxin 2,4D increases TOR-GUS expression in roots [127]. Biochemical experiments in *A. thaliana* cell cultures indicated that the auxin NAA triggers the association of TOR with polysomes and S6K phosphorylation, whereas the asTORi Torin1 has opposite effects [109]. Further experiments with seedlings indicated that the NAA-induced TOR association with polysomes is correlated with an increased abundance of mRNA harboring upstream open reading frames (uORFs) in their leader sequences. This is dependent on the eukaryotic Initiation Factor 3h (eIF3h), which is phosphorylated in a TOR sensitive manner after auxin treatment [109]. Among those uORF containing mRNA regulated by TOR, the auxin response factors ARF3, ARF5, ARF6, and ARF11 mRNAs were found, suggesting that TOR can also transmit the auxin response by regulating the translation of uORF containing mRNAs. This mechanism could occur in root meristems, where ARF5, ARF6, and ARF11 are expressed [128].

**Figure 6:**
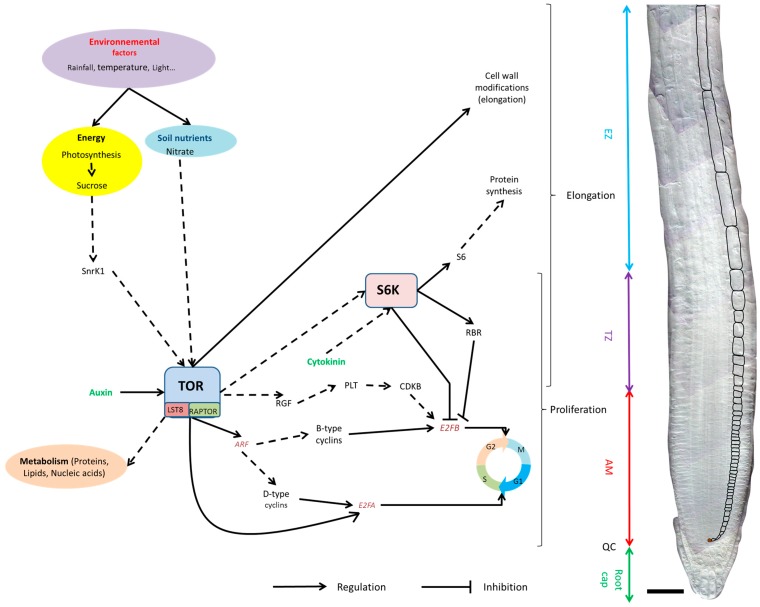
Proposed model of TOR involvement in the regulation of proliferation and growth in the primary root. Plane arrows indicate a proven regulation, whereas doted arrows represent hypothesis that have not been verified yet or which are based on experiments made on other tissues (see main text for details). Bar 50 µm.

Finally, it appears that the TOR pathway plays a major role in root development, regulating the cell cycle genes necessary for proliferation in the meristem. Moreover, TOR plays a role in cell expansion regulating genes involved in cell wall formation and loosening. Thus, TOR seems to regulate the main cellular processes driving growth (Figure 6). Beyond that, TOR might integrate nutrient and hormonal signaling, coordinating and modulating cell proliferation and expansion in response to environmental stimuli. Moreover, TOR seems to be at the intersection of major metabolic pathways, regulating synthesis, recycling, and degradation of the building blocks required for cell growth.

## 4. Conclusions

There is now clear evidence from different research groups that TOR is a central regulator of cell proliferation and cell expansion in the primary roots. Several reports also allowed us to propose a model of how TOR is integrated to the hormonal signals orchestrating the cell cycle and cell growth along the longitudinal axis. However, we are still far from understanding the interactions between TOR and the known proteins involved in this network. Particularly, further work should focus on more precise developmental contexts to identify which members of the TOR pathway are interacting in specific developmental zones, as has been done for several years with RBR. For example, a more precise localization of LST8, RAPTOR, and S6K is required. The organization of the primary roots presented in this review is an ideal system for this purpose. Indeed, it is clear that TOR has different activities depending on the cells in which it is expressed, as illustrated by the fact that it is expressed in both quiescent cells and highly proliferating cells. Pharmacological approaches using the recently developed ATP competitive TOR inhibitors will certainly be essential for future functional studies as they allow reversible modulation of TOR activity in a precise and timely manner. The important models and the molecular biology tools developed since the first work on *A. thaliana* roots in the early 1990s, and briefly presented here, will also be essential for putting the TOR pathway into a developmental perspective in plants. The extensive knowledge of the TOR pathway in yeast and animals, which is also the result of 20 years of intensive research, which has been introduced here, is also bringing new ideas to plant biologists. For example, identifying the subcellular localization and potential new targets of plant TOR as has been done in mammals and in yeast. Indeed, in both yeast and mammalian cells, TORC1 has been localized mainly to the vacuole/lysosome [129]. Furthermore, quantitative phosphoproteomic approaches in yeast have led to the discovery of new TOR pathway targets involved in nucleotide synthesis [130]. However, plant specific functions of the TOR pathways will also need to be investigated in order to shed light on the evolution of this central cellular pathway in photosynthetic organisms.
